# Acute desaturation in intubated patients

**DOI:** 10.1186/cc9577

**Published:** 2011-03-11

**Authors:** P Myrianthefs, C Kouka, E Giannelou, E Evodia, G Baltopoulos

**Affiliations:** 1 School of Nursing, Athens University, Kifissia, Greece; 2 School of Nursing, Athens University, 'Agioi Anargyroi' Hospital, Kifissia, Greece

## Introduction

The purpose of the study was to record the incidence, the etiology and management of acute desaturation (AD) in intubated critically ill ICU patients.

## Methods

We collected demographics of the patients developing AD defined as a documented fall in SaO_2 _(> 3%) in combination with clinical signs of respiratory distress requiring medical intervention. Etiology of AD was investigated by clinical evaluation, ABG analysis and chest X-ray. Numerical data are presented as mean (SEM) or median.

## Results

We included 57 patients (37 men) admitted to our ICU within 6 months of mean age 54.4 (2.7) and mean ICU stay of 25.9 (5.7) days. We recorded 42 episodes of AD in 19 patients (33%). Mean age was 51.4 (3.8), mean ICU stay 51.1 (15.3) days and illness severity APACHE II 20.8 (1.6), SAPS II 52.2 (3.3) and SOFA 9.2 (0.8). The incidence was one episode per 30 ventilator-days or one every 4.3 days, corresponding to 2.3 (1.1) episodes per patient. Mean fall in SaO_2 _was 5%, in PaO_2 _44 mmHg and in PaO_2_/FiO_2 _113. Eight episodes developed while on T-piece due to atelectasis/secretion retension (6) or respiratory muscle fatigue (2). The remaining episodes developed in patients under sedation: atelectasis/secretion retention (10), pulmonary edema (6), fever/SIRS (5), occlusion/displacement of endotracheal tube (5), patient-ventilator asychrony (4), bronchospasm (2), patient transfer (1) and pneumothorax (1). Management included FiO_2 _increase (53.5%), physiotherapy/bronchial toilet/patient poisoning (39.5%), change in ventilator mode (23.3%), PEEP increase (23.3%), drugs (sedation, diuresis, bronchodilators, 16.2%), change in respiratory rate (11.6%), use of Ambu bag (4.6%), reintubation (2.3%), insertion of chest tube (2.3%) and other measures (11.6%). Most patients required at least two interventions. Patients developing AD had significantly higher (*P *< 0.05) SAPS II (median 54 vs. 42), SOFA (9 vs. 6) scores and ICU stay (41 vs. 8). None of the episodes had fatal outcome. Most common hours for developing AD were 07.00, 14.00 and 23.00.

## Conclusions

AD is a common medical emergency condition requiring prompt interventions. One over three patients developed at least two episodes of AD corresponding to one episode per 4.3 days. The most common etiology is atelectasis and secretion retention.

